# Neurotrophin and Trk expression by cells of the human lamina cribrosa following oxygen-glucose deprivation

**DOI:** 10.1186/1471-2202-5-51

**Published:** 2004-12-03

**Authors:** Wendi S Lambert, Abbot F Clark, Robert J Wordinger

**Affiliations:** 1 Department of Cell Biology and Genetics, University of North Texas Health Science Center at Fort Worth, Fort Worth, TX, USA; 2 Glaucoma Research, Alcon Research, Ltd., Fort Worth, TX, USA

## Abstract

**Background:**

Ischemia within the optic nerve head (ONH) may contribute to retinal ganglion cell (RGC) loss in primary open angle glaucoma (POAG). Ischemia has been reported to increase neurotrophin and high affinity Trk receptor expression by CNS neurons and glial cells. We have previously demonstrated neurotrophin and Trk expression within the lamina cribrosa (LC) region of the ONH. To determine if ischemia alters neurotrophin and Trk protein expression in cells from the human LC, cultured LC cells and ONH astrocytes were exposed to 48 hours of oxygen-glucose deprivation (OGD). Also cells were exposed to 48 hours of OGD followed by 24 hours of recovery in normal growth conditions. Cell number, neurotrophin and Trk receptor protein expression, neurotrophin secretion, and Trk receptor activation were examined.

**Results:**

Cell number was estimated using an assay for cell metabolism following 24, 48 and 72 hours of OGD. A statistically significant decrease in LC and ONH astrocyte cell number did not occur until 72 hours of OGD, therefore cellular protein and conditioned media were collected at 48 hours OGD. Protein expression of NGF, BDNF and NT-3 by LC cells and ONH astrocytes increased following OGD, as did NGF secretion. Recovery from OGD increased BDNF protein expression in LC cells. In ONH astrocytes, recovery from OGD increased NGF protein expression, and decreased BDNF secretion. Trk A expression and activation in LC cells was increased following OGD while expression and activation of all other Trk receptors was decreased. A similar increase in Trk A expression and activation was observed in ONH astrocytes following recovery from OGD.

**Conclusions:**

In vitro conditions that mimic ischemia increase the expression and secretion of neurotrophins by cells from the ONH. Increased Trk A expression and activation in LC cells following OGD and in ONH astrocytes following recovery from OGD suggest autocrine/paracrine neurotrophin signaling could be a response to ONH ischemia in POAG. Also, the increase in NGF, BDNF and NT-3 protein expression and NGF secretion following OGD also suggest LC cells and ONH astrocytes may be a paracrine source of neurotrophins for RGCs.

## Background

Glaucoma is an optic neuropathy defined by characteristic optic nerve head and associated visual field changes. Nearly 67 million people worldwide are believed to have glaucoma, including an estimated 2.2 million in the USA [1,2]. Primary open-angle glaucoma (POAG) is the most common form of glaucoma accounting for virtually half of all cases [3]. The visual field changes associated with POAG are due to the loss of retinal ganglion cells (RGCs), which is proposed to occur via apoptosis [4,5]. There is evidence that ischemia contributes to RGC loss in glaucoma. Abnormal optic nerve head (ONH) and retinal blood flow has been observed in glaucoma, and retinal ischemia results in RGC loss [6-11]. In addition, excitotoxicity due to elevated glutamate levels, which occurs following ischemia, can cause RGC death [12-16]. However, not all cellular responses to ischemia are deleterious. The expression of "protective factors", including neurotrophins (NTs), by neurons and glia within the CNS has been shown to increase following ischemia [17-19].

Neurotrophins are polypeptide growth factors involved in the development and maintenance of neurons, as well as non-neuronal cells. Included in this family of trophic factors are nerve growth factor (NGF), brain-derived neurotrophic factor (BDNF), neurotrophin-3 (NT-3) and neurotrophin 4 (NT-4). Neurotrophin signaling occurs via two types of receptors including (a) tyrosine kinase high affinity Trk receptors and (b) the low affinity p75 receptor [20]. The Trk receptors include Trk A, Trk B and Trk C that bind NGF, BDNF and NT-4, and NT-3 respectively [21]. Truncated isoforms of Trk B and Trk C that lack the tyrosine kinase domain have been identified and their function at this time is unknown, although there is evidence that these receptors interfere with full-length trk signaling through ligand sequestration [22-24].

In addition to their localization at axon terminals, Trk receptors have been localized at neuronal cell bodies, at dendritic projections, and along axons [25-27]. Discrete signaling pathways can be activated and distinct biological responses elicited in neurons depending on the location of Trk receptor stimulation indicating neurons respond not only to retrograde NT sources, but also to paracrine and autocrine sources [28-31]. The lamina cribrosa (LC) region of the optic nerve head (ONH) is composed of connective tissue plates that align to form a sieve-like structure that guides and protects RGC axons as they exit the eye to form the optic nerve. Two major cell types have been isolated from the human LC and include ONH astrocytes and LC cells [32-35]. We have previously demonstrated the expression of NTs and their Trk receptors by cells isolated from the human LC [36]. We also reported that mRNA and protein for the low affinity p75 receptor are not expressed by cells isolated from the human ONH. In addition we have shown that LC cells and ONH astrocytes can respond to exogenous NTs via Trk phosphorylation resulting in cell proliferation and secretion of NTs [37]. Due to the proximity of LC cells and ONH astrocytes to RGC axons within the LC, these cells could serve as a paracrine source of NTs for RGCs.

The administration of exogenous NTs has been shown to protect neurons from ischemic damage [38,39] suggesting endogenous NT sources could also be neuroprotective. A recent report by Rudzinski et al. demonstrated that ocular hypertension increased NGF and BDNF expression within the retina [40]. Furthermore, Trk A expression in the retina was increased following elevated intraocular pressure (IOP), and this increase was observed in RGCs. Cui et al. observed an increase in Trk A, Trk B and Trk C expression in RGCs following optic nerve injury [41], again suggesting a neuroprotective role for NTs in RGC injury.

In POAG, the laminar plates of the LC are compressed and bow backward from the sclera producing an excavated and exaggerated optic cup [42]. Because capillaries within the LC are located in the connective tissue plates [43], elevated IOP would likely compromise the blood flow to the LC. Ischemic insults during the progression of POAG could increase the expression and/or secretion of NTs from LC cells and ONH astrocytes, thereby increasing paracrine and/or autocrine NT signaling within the LC.

Given that ischemia can be a component of ocular hypertension, we used oxygen-glucose deprivation (OGD) as an acute model of in vitro ischemia and examined the expression of NTs and their receptors by cultured LC cells and ONH astrocytes following OGD. We are aware that the model used (oxygen and glucose deprivation) is not a perfect model for what occurs in the glaucomatous optic nerve head. However, this acute model was an attempt to mimic the end results of a chronic condition, and as a "first step" we felt this model was adequate for examining the response to injury in these cell types.

## Results

### Cell number following oxygen-glucose deprivation

The LC and ONH astrocyte cell lines used in this study have been previously characterized and described [36]. Preliminary studies examining cell survival following anoxia, hypoxia, hypoglycemia or serum withdrawl demonstrated LC cells and ONH astrocytes were resistant to hypoxia and serum withdrawl alone (data not shown). To approach what is occurring in vivo, we used the more acute oxygen-glucose deprivation (OGD) model to examine NT and trk expression, and NT signaling in cells from the ONH.

**Figure 1:**
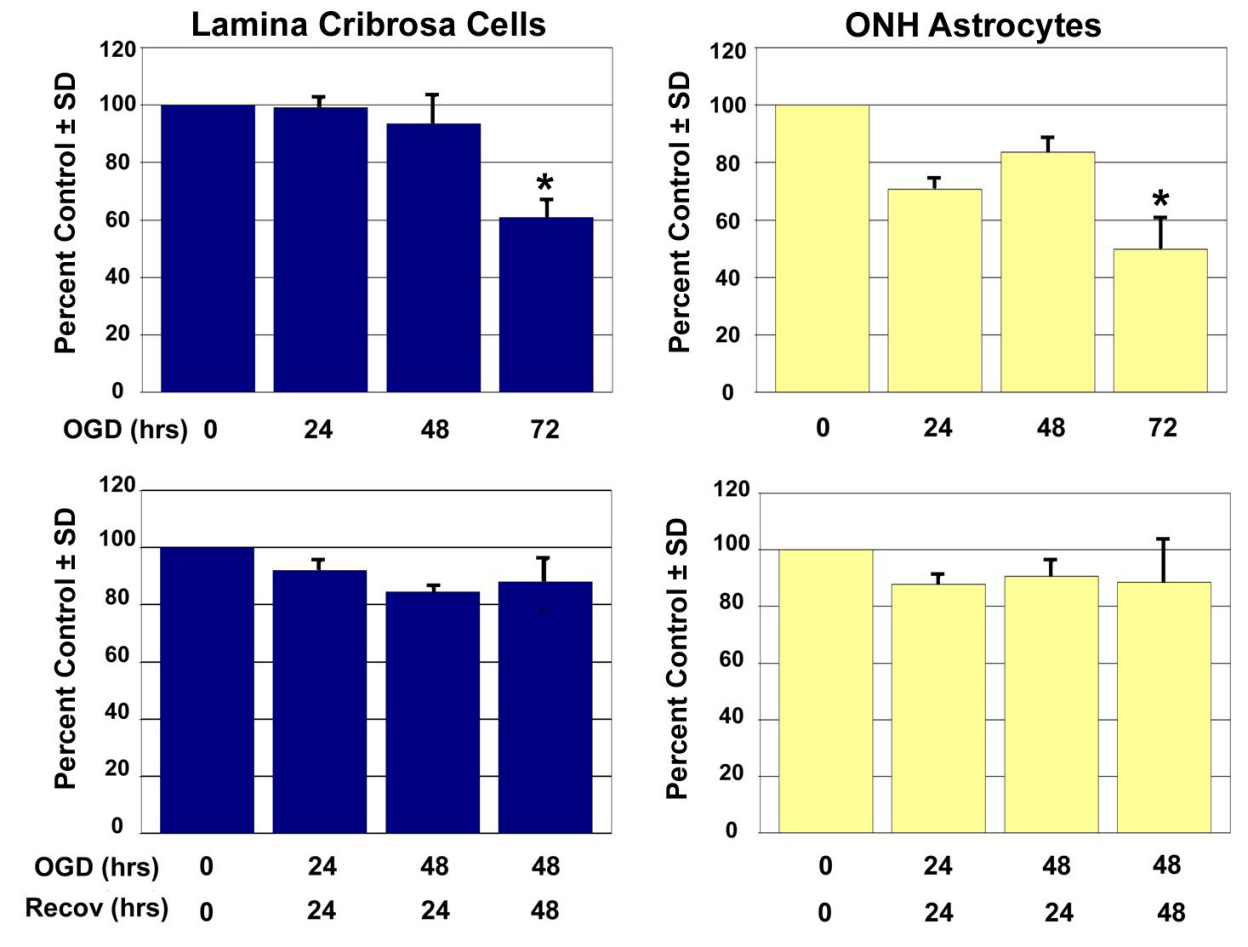
**Cell metabolism/cell number following exposure to and recovery from Oxygen-Glucose Deprivation**. Lamina cribrosa cells and ONH astrocytes were exposed to oxygen-glucose deprivation (OGD), recovery following OGD or control conditions for the time points indicated. Three LC cell lines and three ONH astrocyte cell lines were assayed per experiment. A standard curve was generated for each cell line in order to calculate cell metabolism/cell number. Data shown as percent of the control and represents the mean ± SEM (n = 3). * indicates p < 0.008 (one way analysis of variance [ANOVA] followed by validation using Student-Newman-Keuls tests).

To determine an exposure of OGD that would result in cellular changes while allowing a majority of cells to remain viable, we examined cell number at various time points of OGD using an assay that estimates cell number based on cell metabolism. The oxygen level within the anoxic incubator was determined to be below detectable levels as measured using an Oxygen Test Kit (Bacharach Inc., Pittsburgh, PA). Lamina cribrosa and ONH astrocyte cell metabolism/cell number following OGD is shown in Figure 1. Lamina cribrosa cell metabolism/cell number did not decrease significantly until 72 hours OGD exposure. At this time point a 40% decrease in LC cell metabolism/cell number was observed. A 20% decrease in LC cell metabolism/cell number was observed following 24 or 48 hours recovery when compared to the controls. ONH astrocyte cell metabolism/cell number also decreased 20–30% following 24–48 hours OGD. A statistically significant 50% decrease in cell metabolism/cell number was observed after 72 hours of OGD. Recovery increased ONH astrocyte cell metabolism/cell number slightly when compared to OGD. We chose to collect protein and conditioned media following (a) 48 hours exposure to in vitro ischemia and (b) 48 hours in vitro ischemia plus 24 hours recovery. We chose these time points since greater than 80% of LC cells and ONH astrocytes were present indicating that a majority of cells were viable.

### Neurotrophin protein expression following oxygen-glucose deprivation

**Figure 2:**
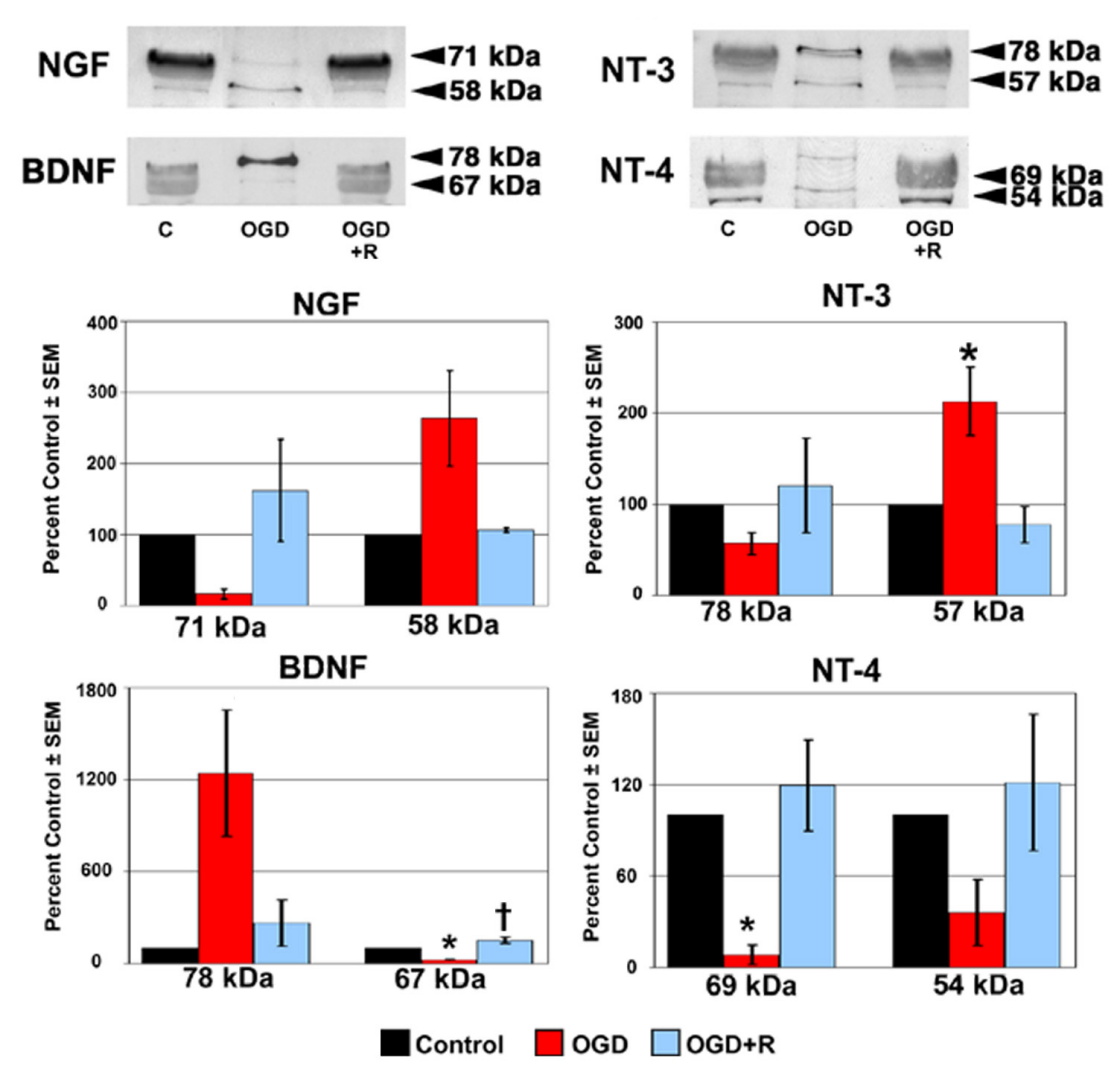
**NT protein expression in LC cells following exposure to and recovery from Oxygen-Glucose Deprivation**. Lamina cribrosa cells were exposed to OGD for 48 hours (OGD) or to 48 hours OGD followed by recovery for 24 hours (OGD+R). Cells exposed to growth media and 95% air/5% CO_2 _for 48 hours served as controls (C). Cell lysate was separated by SDS-PAGE and the density of unsaturated bands was measured for each blot. A representative blot for each NT is shown. Band density reported as mean percent of the control ± SEM (n = 3) is shown graphically beneath the blots. * indicates p < 0.016 for OGD compared to C and OGD+R; † indicates p < 0.016 for OGD+R compared to C (one way analysis of variance [ANOVA] followed by validation using Student-Newman-Keuls tests).

**Figure 3:**
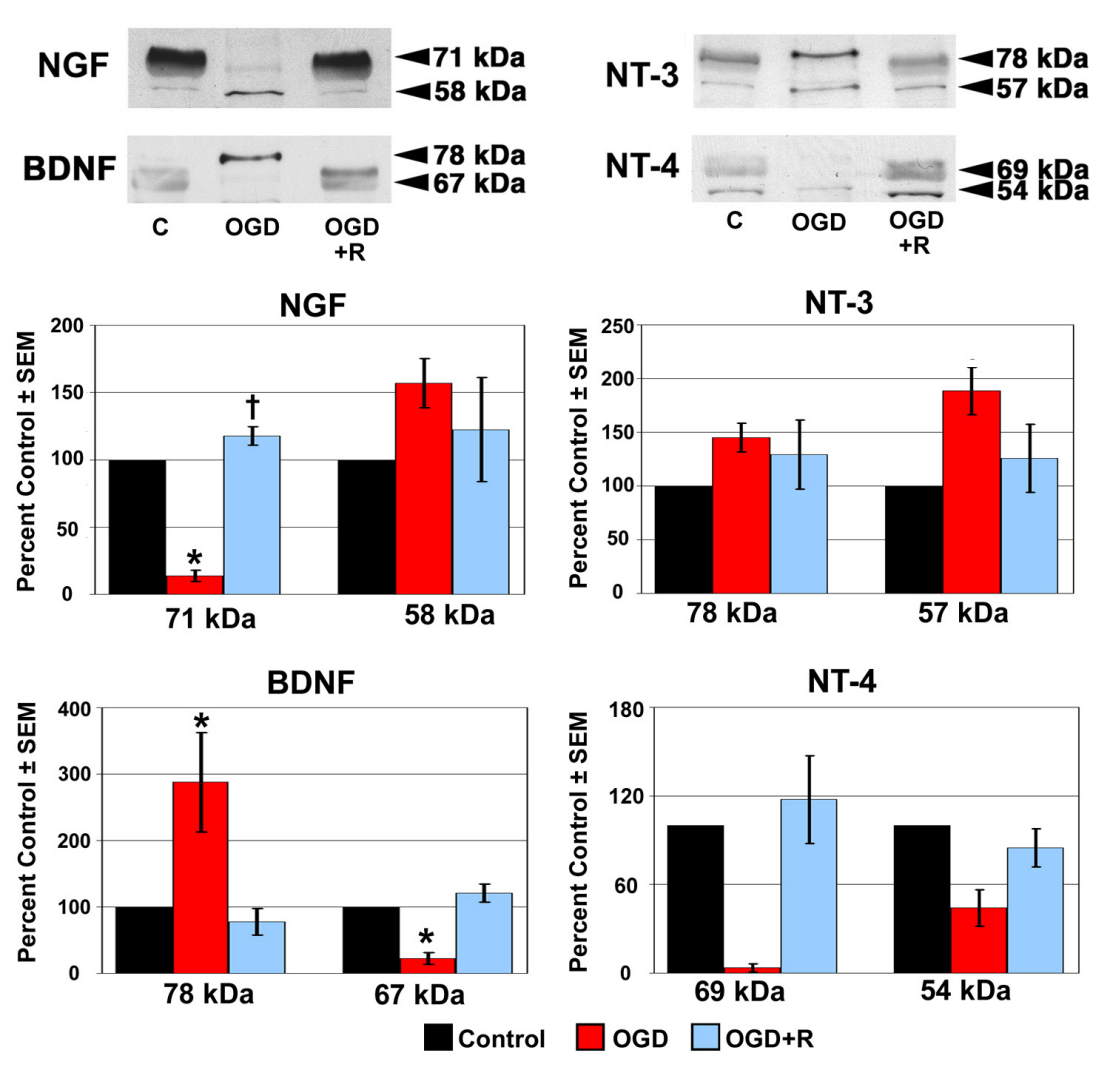
**NT protein expression in ONH astrocytes following exposure to and recovery from Oxygen-Glucose Deprivation**. ONH astrocytes were exposed to OGD for 48 hours (OGD) or to 48 hours OGD followed by recovery for 24 hours (OGD+R). Cells exposed to growth media and 95% air/5% CO_2 _for 48 hours served as controls (C). Cell lysate was separated by SDS-PAGE and the density of unsaturated bands was measured for each blot. A representative blot for each NT is shown. Band density reported as mean percent of the control ± SEM (n = 3) is shown graphically beneath the blots. * indicates p < 0.016 for OGD compared to C and OGD+R; † indicates p < 0.016 for OGD+R compared to C (one way analysis of variance [ANOVA] followed by validation using Student-Newman-Keuls tests).

The expression of NT protein in LC cells and ONH astrocytes following OGD is shown in Figures 2 and 3. Multiple isoforms for all four NTs were observed in LC cells and ONH astrocytes. These isoforms most likely represent proneurotrophins, premature forms of the NTs that have recently been shown to possess biological activity [44]. A representative blot for each NT is shown. Band density reported as mean percent of the control ± SEM of three cell lines is shown graphically beneath the blots. Western blots for NTs and trks were stripped and re-probed for β-actin to ensure equal loading. Expression of β-actin in LC cells and ONH astrocytes was not significantly influenced by OGD or recovery from OGD. The overall average β-actin band density reported as a percent of the control ± SEM for LC cells was 108% ± 7 following OGD exposure and 96% ± 3 following recovery from OGD. Similar changes were observed in ONH astrocytes (109% ± 8 following OGD and 107% ± 7 following recovery from OGD).

As seen in Figure 2, the overall trend following 48 hours of OGD was a decrease in NT protein expression by LC cells. The exceptions to this trend were NGF (58 kDa), BDNF (78 kDa), and NT-3 (57 kDa), of which only NT-3 (57 kDa) demonstrated a statistically significant increase in protein expression following OGD. Of the remaining NT isoforms, BDNF (67 kDa) and NT-4 (69 kDa) demonstrated a statistically significant decrease in expression following OGD. A recovery period of 24 hours in growth media and an aerobic environment following 48 hours OGD resulted in elevated NT protein expression by LC cells, however only BDNF (67 kDa) expression levels were statistically significant.

Following 48 hours of OGD, ONH astrocytes (Figure 3) demonstrated increased protein expression of NGF (58 kDa), NT-3 (57 kDa) and BDNF (78 kDa); only BDNF (78 kDa) was statistically significant. In addition, the expression of NGF (71 kDa) and BDNF (67 kDa) was decreased to a statistically significant level following OGD. Increased NT protein expression was the trend in ONH astrocytes allowed to recover from OGD for 24 hours in growth media and an aerobic environment. The exceptions were BDNF (78 kDa) and NT-4 (54 kDa), which were slightly lower than control levels. Of the NT isoforms where protein expression increased during recovery, only NGF (71 kDa) demonstrated a statistically significant increase. Western blot densitometry results for LC cells and ONH astrocytes are summarized in Table 1.

### Trk receptor protein expression following oxygen-glucose deprivation

**Figure 4:**
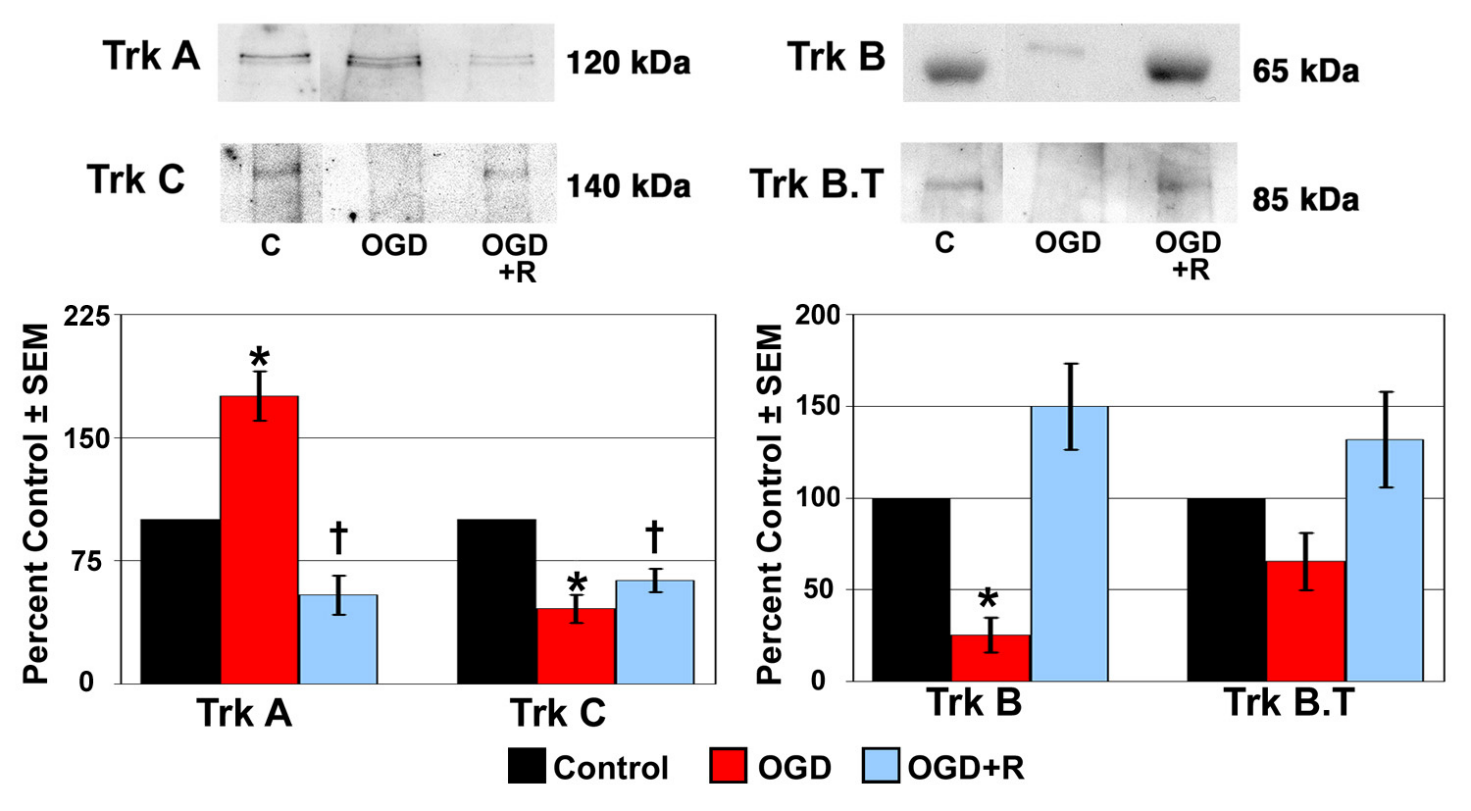
**Trk receptor protein expression in LC cells following exposure to and recovery from Oxygen-Glucose Deprivation**. Lamina cribrosa cells were exposed to OGD for 48 hours (OGD) or to 48 hours OGD followed by recovery for 24 hours (OGD+R). Cells exposed to growth media and 95% air/5% CO_2 _for 48 hours served as controls (C). Cell lysate was separated by SDS-PAGE and the density of unsaturated bands was measured for each blot. A representative blot for each Trk receptor is shown. Band density reported as mean percent of the control ± SEM (n = 3) is shown graphically beneath the blots. * indicates p < 0.016 for OGD compared to C and OGD+R; † indicates p < 0.016 for OGD+R compared to C (one way analysis of variance [ANOVA] followed by validation using Student-Newman-Keuls tests). Trk B.T; truncated Trk B.

Figure 4 represents the expression of Trk receptor protein in LC cells following OGD or recovery from OGD. Data are presented as described in the previous section. Protein expression of Trk B and Trk C receptors decreased to a statistically significant level in LC cells following 48 hours of OGD. In contrast, a statistically significant increase in the expression of Trk A protein was observed following exposure to OGD. Recovery from OGD resulted in a statistically significant decrease in Trk A and Trk C expression, while Trk B and truncated Trk B protein expression was elevated, but not to a statistically significant level.

**Figure 5:**
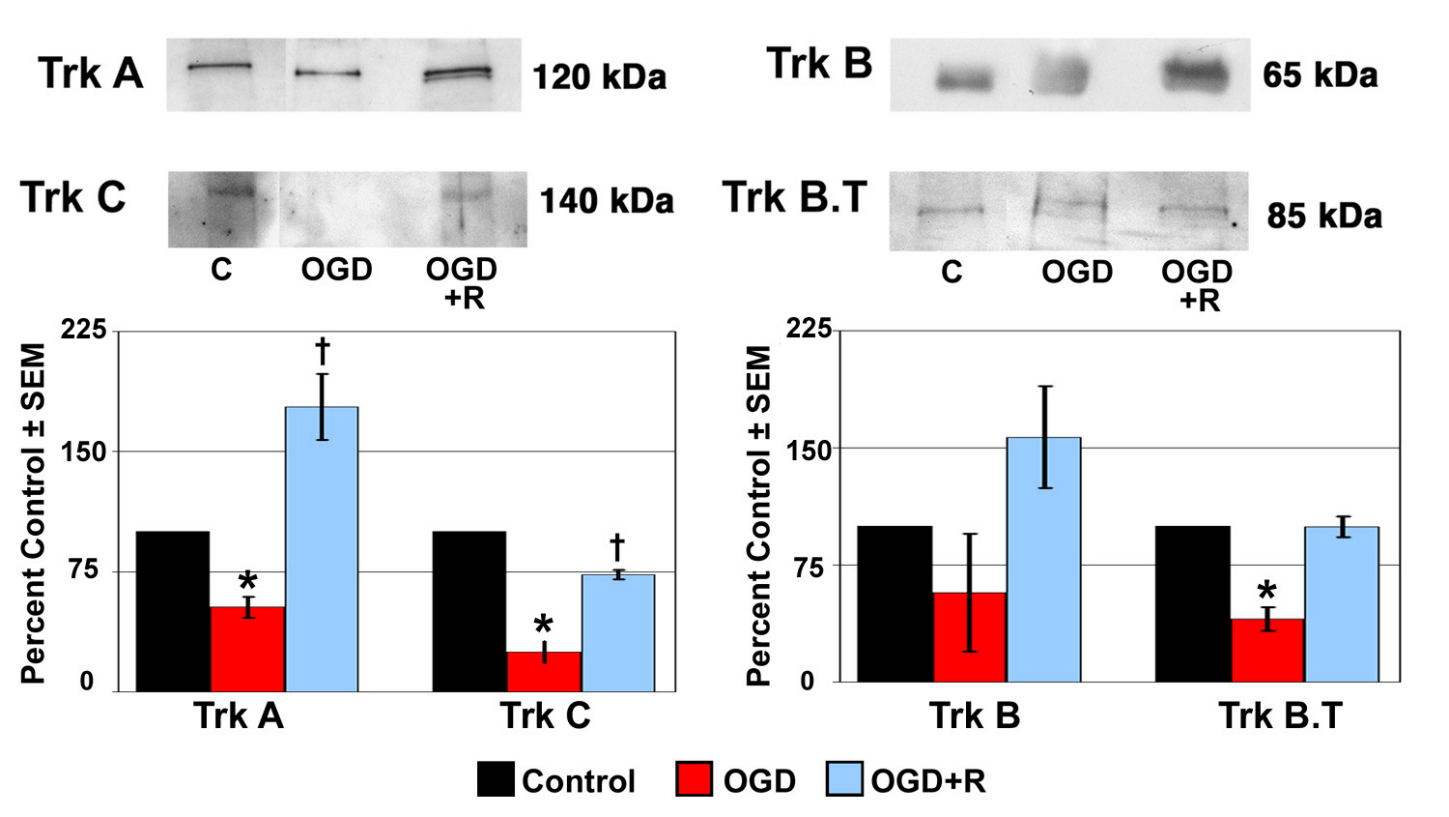
**Trk receptor protein expression in ONH astrocytes following exposure to and recovery from Oxygen-Glucose Deprivation**. ONH astrocytes were exposed to OGD for 48 hours (OGD) or to 48 hours OGD followed by recovery for 24 hours (OGD+R). Cells exposed to growth media and 95% air/5% CO_2 _for 48 hours served as controls (C). Cell lysate was separated by SDS-PAGE and the density of unsaturated bands was measured for each blot. A representative blot for each Trk receptor is shown. Band density reported as mean percent of the control ± SEM (n = 3) is shown graphically beneath the blots. * indicates p < 0.016 for OGD compared to C and OGD+R; † indicates p < 0.016 for OGD+R compared to C (one way analysis of variance [ANOVA] followed by validation using Student-Newman-Keuls tests). Trk B.T; truncated Trk B.

The expression of Trk receptor protein by ONH astrocytes following OGD and recovery from OGD is shown in Figure 5. A statistically significant decrease in the expression of Trk A, Trk C and truncated Trk B protein by ONH astrocytes was observed following OGD. Trk B protein expression was also decreased, but not to a significant level. Recovery from OGD increased Trk C and truncated Trk B receptor protein expression in ONH astrocytes compared to OGD alone. However, Trk C expression was still decreased to a statistically significant level. Trk A and Trk B protein expression was increased in ONH astrocytes allowed to recover from OGD, although only Trk A expression was statistically significant.

### Phosphorylated Trk receptor protein expression following oxygen-glucose deprivation

**Figure 6:**
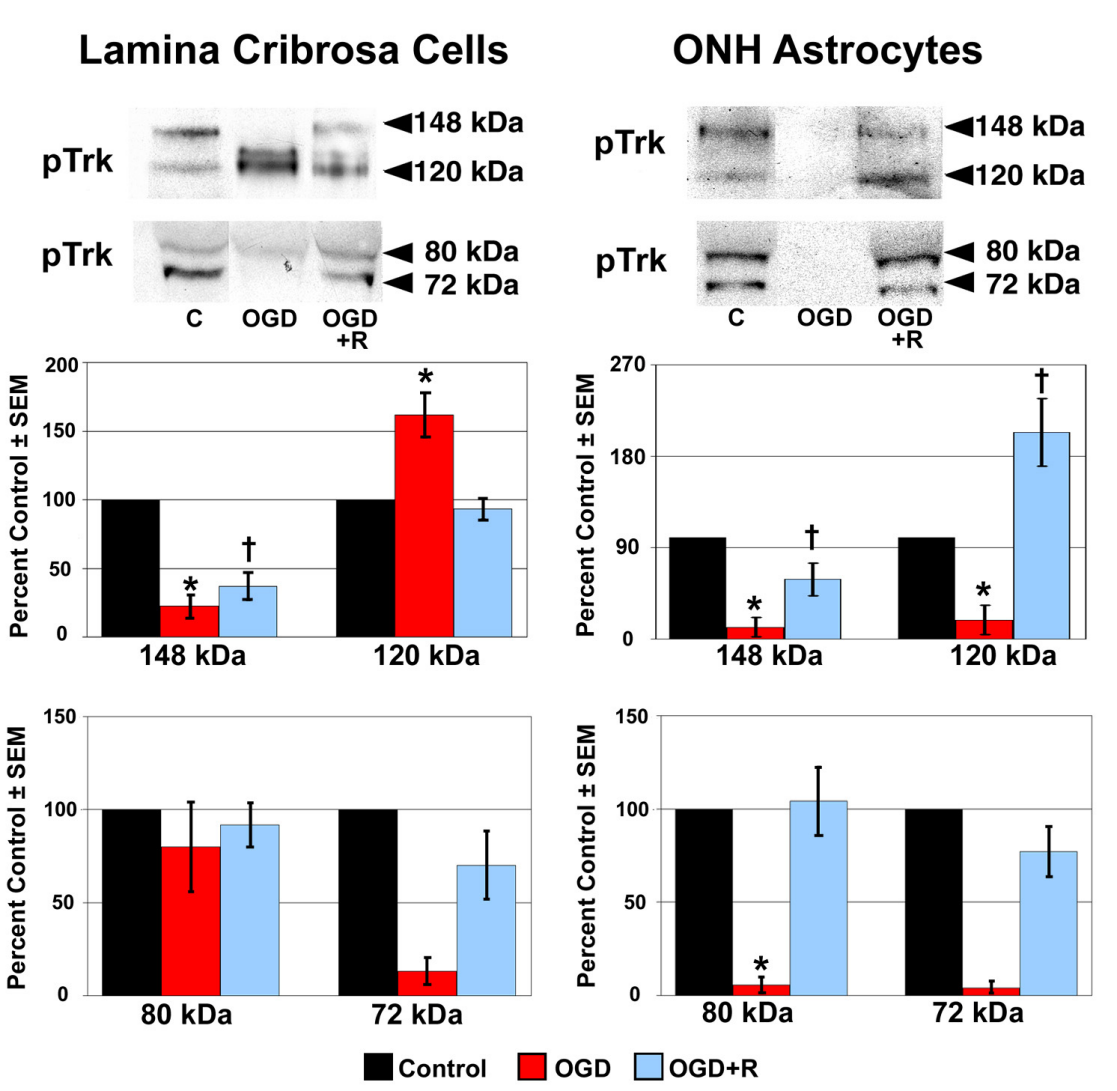
**Phosphorylated Trk protein expression following exposure to and recovery from Oxygen-Glucose Deprivation**. Lamina cribrosa cells and ONH astrocytes were exposed to OGD for 48 hours (OGD) or to 48 hours OGD followed by recovery for 24 hours (OGD+R). Cells exposed to growth media and 95% air/5% CO_2 _for 48 hours served as controls (C). Cell lysate was separated by SDS-PAGE and the density of unsaturated bands was measured for each blot. A representative blot for each phospho-Trk receptor isoform is shown. Band density reported as mean percent of the control ± SEM (n = 3) is shown graphically beneath the blots. * indicates p < 0.016 for OGD compared to C and OGD+R; † indicates p < 0.016 for OGD+R compared to C (one way analysis of variance [ANOVA] followed by validation using Student-Newman-Keuls tests).

The expression of phosphorylated Trk receptor protein following OGD and recovery from OGD is shown in Figure 6. Data are presented as described above. Trk phosphorylation indicates the activation of Trk receptors following NT binding. The antibody used was a phospho-pan Trk antibody that recognizes phosphorylated forms of Trk A, Trk B and Trk C receptors. Four phospho-Trk isoforms (148 kDa, 120 kDa, 80 kDa, and 72 kDa) were detected in LC cell and ONH astrocyte controls. The 120 kDa and 72 kDa phospho-Trk isoforms most likely represent phosphorylated Trk A and Trk B respectively [37]. The remaining phospho-Trk isoforms could represent phosphorylated forms of Trk A, B or C.

Overall, exposure to OGD resulted in decreased phospho-Trk protein expression by LC cells and ONH astrocytes. Expression of the 148 kDa phospho-trk isoform was decreased to a statistically significant level in both LC cells and ONH astrocytes, as was the 120 kDa and 80 kDa isoforms in ONH astrocytes. The only increase in phospho-Trk receptor protein expression following OGD was observed in LC cells. Protein expression of the 120 kDa phospho-Trk receptor isoform was increased 60% over the controls in LC cells, which was statistically significant.

Recovery from OGD resulted in increased protein expression of phospho-Trk receptor isoforms in LC cells and ONH astrocytes when compared to OGD alone, with the exception of the 120 kDa isoform in LC cells, which decreased toward control levels. Although its expression increased compared to OGD alone, expression of the 148 kDa isoform was still decreased to a significant level. Interestingly, the 120 kDa phospho-trk isoform was again the only isoform whose expression was increased above control levels. This 100% increase was observed in ONH astrocytes following recovery from OGD and was statistically significant.

### Neurotrophin secretion following oxygen-glucose deprivation

**Figure 7:**
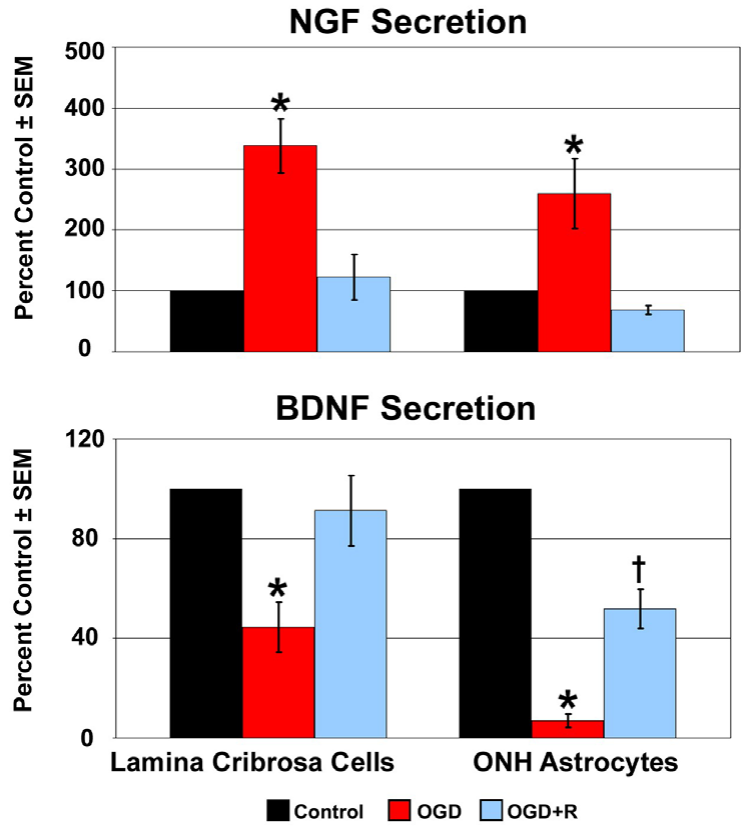
**NT secretion following exposure to and recovery from Oxygen-Glucose Deprivation**. Lamina cribrosa cells and ONH astrocytes were exposed to OGD for 48 hours (OGD) or to 48 hours OGD followed by recovery for 24 hours (OGD+R). NGF and BDNF secretion was determined by immunoassay of concentrated conditioned media from three LC cell lines and three ONH astrocyte cell lines. A NT standard was included in each assay and was used to generate a standard curve. Data are reported as percent of the control ± SEM (n = 3). * indicates p < 0.016 for OGD compared to C and OGD+R; † indicates p < 0.016 for OGD+R compared to C (one way analysis of variance [ANOVA] followed by validation using Student-Newman-Keuls tests).

The secretion of NGF and BDNF following OGD and recovery from OGD is shown in Figure 7. Data are presented as mean percent of the control ± SEM of three LC and three ONH astrocyte cell lines. The secretion of NGF by LC cells and ONH astrocytes was increased over 200% and 150% following OGD, respectively. This increase in NGF secretion by cultured cells of the human LC was statistically significant. In contrast, BDNF secretion by both cell types was decreased 60 to 90% following OGD, which was statistically significant. Although recovery from OGD returned NGF and BDNF secretion by LC cells toward controls levels, a statistically significant decrease in BDNF secretion by ONH astrocytes was observed. The secretion of NT-3 or NT-4 was not detected by either cell type under any condition.

## Discussion

In this study we examined the protein expression of NTs and Trk receptors by LC cells and ONH astrocytes following OGD, an in vitro model of ischemia. Lamina cribrosa cell and ONH astrocyte responses to OGD and recovery from OGD are summarized in Tables 2 and 3. Lamina cribrosa cells and ONH astrocytes increased NGF, BDNF and NT-3 protein expression following OGD. NGF secretion by these cells was also increased by OGD at the time points tested. Exposure to OGD decreased Trk protein expression and activation in LC cells and ONH astrocytes, with the exception of Trk A in LC cells. Recovery from OGD resulted in most NT and Trk receptor protein expression returning toward control levels in LC cells and ONH astrocytes. However, Trk A expression and activation in ONH astrocytes remained significantly elevated over control levels. Overall, this study suggests that paracrine and/or autocrine NT signaling is stimulated in cells from the ONH following an ischemic insult. Alternatively, NT release by ONH cells may act to protect RGCs from ischemic injury.

Ischemia due to elevated IOP during POAG may cause changes within the ONH and contribute to RGC loss [6-11]. As a response to ischemia, neurons and glia have been shown to increase the expression of NTs [17-19]. Local NT sources for RGCs within the retina and LC could potentially protect these neurons during periods of ONH ischemia in POAG. The recent report of Rudzinski et al. [40] is of interest as they demonstrated an up-regulation of both NGF and Trk A after 7 days of ocular hypertension, and a sustained up-regulation of BDNF after 28 days elevated IOP. We have previously demonstrated NT expression and secretion by LC cells and ONH astrocytes [36]. The LC region of the ONH and the cells that reside there may be subject to ischemic injury during the progression of POAG. Therefore, we examined cell number as determined by cell metabolism following OGD to determine if LC cells and ONH astrocytes could survive ischemic insult. Lamina cribrosa cell metabolism/cell number remained within 15% of the controls until 72 hours of OGD, whereas ONH astrocyte cell metabolism/cell number decreased 20–30% over the same exposure time. Lamina cribrosa and ONH astrocyte cell metabolism/cell number returned to within 20% of the controls following recovery from OGD. These results suggest LC cells and ONH astrocytes can survive ischemic injury and therefore could be a potential source of neuroprotective factors.

Neurons, including RGCs, express Trk receptors not only at the axon terminal and cell body, but also along their axons [25-27] suggesting RGCs could bind NTs provided by cells of the ONH. We examined the expression and secretion of NTs by LC cells and ONH astrocytes following OGD to determine if these cells could potentially protect RGCs from ischemic injury. The expression of NGF, BDNF and NT-3 by LC cells and ONH astrocytes increased after exposure to OGD. Examination of NT mRNA levels in LC cells and ONH astrocytes following OGD would determine whether this is due to an up-regulation in expression or to increased processing of proNTs. Interestingly, only NGF secretion by LC cells and ONH astrocytes was increased by OGD. It is possible that BDNF and NT-3 are secreted by LC cells and ONH astrocytes following OGD, but at time points other than those examined or at lower detection levels. Overall, the responses to OGD by LC cells and ONH astrocytes with respect to the expression of NTs appeared favorable to RGCs. Neurotrophin expression was increased in both cell types, as was NGF secretion. Thus it appears LC cells and ONH astrocytes could provide RGCs with neuroprotective factors following ischemic injury.

The expression of Trk receptors (both full length and truncated) by LC cells, ONH astrocytes or other cells within the LC could limit NT availability to RGCs [22-24]. Therefore, we examined Trk protein expression and activation in LC cells and ONH astrocytes following OGD. Trk receptor expression was decreased in LC cells and ONH astrocytes following OGD, with the significant exception of Trk A in LC cells. As an indication of NT signaling, we examined the expression of phosphorylated Trk receptors in LC cells and ONH astrocytes following OGD. Phospho-Trk A expression in LC cells was increased after OGD, while all other phospho-Trk expression was decreased. The up-regulation of autocrine NGF signaling in LC cells (e.g. NGF secretion and Trk A expression) could explain the differences observed between LC and ONH astrocyte cell number in following OGD. In a previous report we demonstrated that exogenous NGF increased LC cell number [37]. As LC cells do not express p75, this response is most likely due to Trk A activation. Together these data suggest Trk expression by ONH astrocytes would not interfere with NT availability to RGCs during ischemia. In contrast, increased expression of Trk A in LC cells resulted in increased Trk A activation, implying NGF expression in these cells during ischemia may be self protective rather than protective toward RGCs.

There is evidence that reperfusion following ischemia is actually more detrimental to cells than ischemia itself [45,46]. To determine if cells from the LC could provide RGCs with NT support during reperfusion of the ONH in POAG, we examined NT and Trk receptor expression following recovery from OGD. ONH astrocytes could be a source of NTs for RGCs during reperfusion of the ONH as an increase in NGF protein expression was observed in these cells after recovery from OGD. However, recovery from OGD also increased Trk A expression and activation in ONH astrocytes, implying these cells up-regulate autocrine/paracrine NGF signaling during recovery from an ischemic event. In addition, Trk B receptor expression by LC cells and ONH astrocytes increased after recovery from OGD suggesting NT availability to RGC axons within the LC may be compromised during ONH reperfusion. Based on these results, LC cells and ONH astrocytes would be unable to provide RGCs with NTs during reperfusion of the ONH. Increased NT expression by LC cells and ONH astrocytes during ONH ischemia could protect RGCs from injury; however, as blood flow was restored NT expression by LC cells and ONH astrocytes would decrease to normal levels, leaving RGCs vulnerable to injury. Neurotrophin expression by LC cells and ONH astrocytes may be beneficial to RGCs during ischemia, but may be unable to promote the survival of these neurons during reperfusion.

In conclusion, we have demonstrated that LC cells and ONH astrocytes increase the expression of NGF, BDNF and NT-3 protein following OGD, which may be neuroprotective for RGCs. Neurotrophins expressed by LC cells and ONH astrocytes following OGD or recovery from OGD bind and activate Trk receptors expressed by these cells. Increased NT signaling within LC cells and ONH astrocytes could increase cell survival following ischemic injury, but may compromise RGC survival during reperfusion. Further studies examining the expression of NTs and Trk receptors following hypoxia or transient ischemic insults would provide a better model for ischemic injury in POAG. Using this model, RGCs could be co-cultured with LC cells or ONH astrocytes to determine the neuroprotective effects of NTs during ischemic injury. By better understanding NT signaling within the LC under normal and injurious conditions, new strategies involving these factors could be developed to better treat patients with POAG.

## Conclusions

Lamina cribrosa cells and ONH astrocytes respond to conditions that mimic ONH ischemia by increasing NGF, BDNF and NT-3 protein expression and NGF secretion.

Increased protein expression of Trk receptors and phosphorylated Trk receptors by LC cells and ONH astrocytes following OGD and recovery from OGD respectively suggest paracrine and/or autocrine NT signaling occurs within the ONH following ischemic injury.

Lamina cribrosa cells and ONH astrocytes may be a paracrine source of NGF, BDNF and/or NT-3 for RGCs, especially during ischemic injury within the ONH throughout POAG progression.

## Methods

### Materials

DMEM and fetal bovine serum (FBS) were purchased from HyClone Labs, Logan, UT. The following materials were purchased from Gibco BRL Life Technologies, Grand Island, NY; glucose free DMEM, L-glutamine, penicillin/streptomycin and fungizone (amphotericin B). Costar 96-well plates and Nunc ELISA/EIA 96 well Maxisorp plates were purchased from Fisher Scientific, Pittsburgh, PA. Polyclonal antibodies to Trk A, Trk B, Trk C, truncated Trk B (Trk B.T) and phosphorylated Trk were purchased from Santa Cruz Biotechnology Inc, Santa Cruz, CA. CellTiter 96^® ^Aqueous Non-Radioactive Cell Proliferation Assays and Emax™ ImmunoAssay Systems specific for NGF, BDNF, NT-3 and NT-4 were purchased from Promega Corporation, Madison, WI.

### Lamina cribrosa and ONH astrocyte cell culture

Lamina cribrosa and ONH astrocyte cell lines were obtained from human LC explants from separate donors as described previously [36]. Cells were cultured in Ham's F-10 Media (LC cells, JRH Biosciences, Lenexa, KS) or DMEM (ONH astrocytes) supplemented with 10% FBS, L-glutamine (0.292 mg/ml), penicillin (100 units/ml)/streptomycin (0.1 mg/ml), and amphotericin B (4 μg/ml) as previously described. Cells were passaged using a 0.25% trypsin solution (Sigma-Aldrich, St. Louis, MO). All cultures were maintained in 5% CO_2_/95% air at 37°C and media was changed every 2 to 3 days. Characterization of these cells was performed as described previously [36]. Cells expressing α-smooth muscle actin that did not express glial fibrillary acidic protein (GFAP) were characterized as LC cells [32,33,36]. Cells expressing GFAP and neural cell adhesion molecule (N-CAM) were characterized as ONH astrocytes [34-36]. Both cell types expressed extracellular matrix proteins, such as collagen I, collagen III, collagen IV and elastin [32,33,36]. Adult cell lines from donors whose ages ranged from 39 years to 90 years were used in the following experiments.

### Oxygen-glucose deprivation

Preliminary studies examining cell survival following anoxia, hypoxia, hypoglycemia or serum withdrawl demonstrated LC cells and ONH astrocytes were resistant to hypoxia and serum withdrawl alone (data not shown). To approach what is occurring in vivo, we used the more acute oxygen-glucose deprivation (OGD) model to examine NT and trk expression and NT signaling in cells from the ONH. Preconfluent, age-matched adult LC cells and ONH astrocytes were treated with serum free media for 24 hours. Oxygen-glucose deprivation (OGD) was achieved by culturing LC cells and ONH astrocytes in glucose free serum free DMEM in an anoxic incubator (95% N_2_/5% CO_2_) for 48 hours. The oxygen level within the anoxic incubator was measured using a Oxygen Test Kit (Bacharach Inc., Pittsburgh, PA) and was determined to be below detectable levels. Recovery following OGD was achieved by placing cells in OGD conditions for 48 hours and then allowing them to recover in growth media (Ham's F-10 Media or DMEM plus 10% FBS) and an aerobic environment (95% air/5% CO_2_) for 24 hours. Cells cultured in growth media and an aerobic environment for served as controls.

### Determination of cell number based upon cell metabolism following oxygen-glucose deprivation

Adult LC cells and ONH astrocytes were trypsinized, counted using a hemacytometer and plated into Costar 96-well plates at a density of 1,000 cells/well. Cells were allowed to attach overnight and were then placed in serum free media for 24 hours. Lamina cribrosa cells and ONH astrocyte were exposed to OGD as described above for 24, 48 or 72 hours. Recovery following OGD was achieved by placing cells in OGD conditions for 24 or 48 hours and then allowing them to recover in growth media and an aerobic environment (95% air/5% CO_2_) for 24 hours. Cells cultured in growth media and an aerobic environment for served as controls.

Cell number was estimated using the CellTiter 96^® ^Aq_ueous _Non-Radioactive Cell Proliferation Assay which measures product from a metabolic process to estimate cell number. Following exposure to OGD or recovery from OGD, media was removed and replaced with 100 μl of serum free media. Twenty microliters of MTS/PMS solution was added to each well. Plates were incubated at 95% air/5% CO_2 _at 37°C for 1 hour, at which time the absorbance at 490 nm was read using a SpectraMax^® ^190 microplate reader and Softmax^® ^Pro (Molecular Devices Corporation, Sunnyvale, CA). Metabolically active cells convert MTS into formazan, which is soluble in aqueous solutions. The quantity of the formazan product measured by the amount of absorbance at 490 nm is therefore directly proportional to the number of living cells. Cell metabolism/cell number per well was calculated from a standard curve generated using known amounts of cells per well. A standard curve was generated for each cell line assayed. Three LC cell lines and three ONH astrocyte cell lines were assayed. The entire experiment, including standard curves, was repeated twice. Changes in cell metabolism/cell number following OGD and recovery from OGD were reported as a percent of the control ± SEM.

### Protein extraction and western blot analysis

Cellular protein was collected in lysis buffer modified from Watson et al. [47] [20 mM Tris (pH 7.4), 137 mM NaCl, 1% NP40, 10% glycerol, 48 mM sodium fluoride, 16 mM sodium pyrophosphate, 1 mM PMSF, 20 μM leupeptin, 10 μg/ml aprotinin, and 1 mM sodium orthovanadate (10 μl/ml)]. Protein concentration was measured using the Bio-Rad D_c _Protein Assay System (Bio-Rad Laboratories, Richmond, CA). Cellular lysate (50 μg) was separated on denaturing polyacrylamide gels and then transferred by electrophoresis to nitrocellulose membranes. Blots were processed using primary antibodies and the Western Breeze Chemiluminescent Immunodetection System (Invitrogen, Carlsbad, CA). Blots were then exposed to Hyperfilm-ECL (Amersham, Arlington Heights, IL) for various times depending on the amount of target protein present. The density (O.D. × mm^2^) of unsaturated bands was measured using the Discovery Series scanner and the Diversity One program from pdi (Huntington, NY) and a digital Venturis FP466 computer (Compaq, Houston, TX). Western blots for NTs and trks were stripped and re-probed for β-actin to ensure equal loading.

### Conditioned media and immunoassays

Conditioned media was collected and concentrated using Millipore Centriplus YM-3 Centrifugal Filter Devices (Millipore Corporation, Bedford, MA). Emax™ ImmunoAssay Systems specific for NGF, BDNF, NT-3 and NT-4 (Promega) were performed according to manufacturer's instructions. Conditioned media was added to Nunc ELISA/EIA 96 well Maxisorp plates coated with anti-NT polyclonal antibodies. Secreted NT was detected by treating the plates with the respective NT monoclonal antibody followed by a horseradish peroxidase conjugated secondary antibody. Enzyme substrate was added to generate a color product whose absorbance was read at 450 nm. A NT standard included in each assay was used to generate a standard curve that was used to calculate the amount of secreted NT per well. The amount of secreted NT per sample was normalized to total protein per sample. Samples were assayed in triplicate. Conditioned media from three LC cell lines and three ONH astrocyte cell lines were assayed. Each immunoassay was repeated twice. Changes in NT secretion following OGD and recovery from OGD were reported as a percent of the control ± SEM.

### Statistical analysis

Cell metabolism/cell number, western blot densitometry and immunoassay data were analyzed using one way analysis of variance (ANOVA) followed by validation using Student-Newman-Keuls tests. The MedCalc^® ^statistical package, version 7.4.41 [48] was used for statistical analysis. Significance values were adjusted in accordance with Bonferroni's correction for multiple tests [49].

## Authors' contributions

WL carried out tissue culture, cell proliferation assays, Western blotting, and immunoassays. WL, AC, and RW participated in design of the study, interpretation of the results, and in the writing and revision of the manuscript. All authors read and approved the final manuscript. This study is taken in part from a dissertation submitted to the UNT Health Science Center in partial fulfillment of the requirements for the degree Doctor of Philosophy for WL.

## Figures and Tables

**Table 1:** Summary of western blot densitometry data expressed in percent of the control ± SEM of three cell lines.

	Lamina cribrosa cells		ONH astrocytes	
	OGD	OGD+R	OGD	OGD+R

NGF (71 kDa)	16 ± 7	162 ± 71	14 ± 4	118 ± 7
NGF (58 kDa)	264 ± 67	106 ± 3	157 ± 18	122 ± 39
BDNF (78 kDa)	1244 ± 414	265 ± 152	288 ± 75	78 ± 20
BDNF (67 kDa)	22 ± 6	151 ± 22	22 ± 9	121 ± 13
NT-3 (78 kDa)	57 ± 12	120 ± 52	145 ± 13	129 ± 32
NT-3 (57 kDa)	213 ± 37	77 ± 20	188 ± 22	126 ± 32
NT-4 (69 kDa)	8 ± 7	119 ± 30	4 ± 3	117 ± 30
NT-4 (54 kDa)	32 ± 13	98 ± 35	44 ± 12	85 ± 13
Trk A	175 ± 15	54 ± 12	53 ± 7	178 ± 21
Trk B	25 ± 9	150 ± 24	57 ± 38	157 ± 33
Trk C	46 ± 9	63 ± 7	25 ± 7	73 ± 3
Trk B.T	65 ± 16	132 ± 26	40 ± 8	99 ± 7
pTrk (148 kDa)	23 ± 9	37 ± 10	12 ± 10	59 ± 16
pTrk (120 kDa)	162 ± 16	93 ± 8	19 ± 14	204 ± 19
pTrk (80 kDa)	80 ± 14	92 ± 12	6 ± 4	104 ± 18
pTrk (72 kDa)	13 ± 7	70 ± 18	4 ± 3	77 ± 13

**Table 2:** Lamina cribrosa cell and ONH astrocyte responses to oxygen-glucose deprivation.

	Lamina cribrosa cells	ONH astrocytes
Cell Number	95 % survival at 24 hrs, 85% at 48 hrs	70 % survival at 24 hrs, 80% at 48 hrs
NT Expression	↑ NGF, ↑ BDNF, ↑ NT-3; ↓ NT-4	↑ BDNF, ↑ NT-3; ↓ NGF, ↓ NT-4
Trk Expression	↑ Trk A; ↓ Trk C, ↓ Trk B	↓ Trk A, ↓ Trk C, ↓ Trk B.T
Phospho-Trk Expression	↑ 120 kDa isoform (Trk A); ↓ 148 kDa isoform	↓ all phospho-trk receptors
	isoform	
Secretion of NTs	↑ NGF; ↓ BDNF	↑ NGF; ↓ BDNF

**Table 3:** Lamina cribrosa cell and ONH astrocyte responses to recovery following oxygen-glucose deprivation.

	Lamina cribrosa cells	ONH astrocytes
Cell Number	80 % survival	80 % survival
NT Expression	↑ BDNF; others near control levels	↑ NGF
Trk Expression	↓ Trk A, ↓ Trk C	↑ Trk A
Phospho-Trk Expression	↓ 148 kDa isoform; others near control levels	↑ 120 kDa isoform (Trk A); ↓ 148 kDa isoform
Secretion of NTs	near control levels	↓ BDNF
